# Physical activity in older adults as a predictor of alcohol consumption – a longitudinal analysis of 3133 individuals in the SHARE study

**DOI:** 10.1192/j.eurpsy.2025.2417

**Published:** 2025-03-20

**Authors:** Sabine Weber, Daniel König, Thomas Waldhoer, Brendon Stubbs, Theresa Lichtenstein, Armin Trojer, Lea Sommer, Benjamin Vyssoki, Melanie Trimmel, Fabian Friedrich, Stephan Listabarth

**Affiliations:** 1 Clinical Division of Social Psychiatry, Department of Psychiatry and Psychotherapy, Medical University of Vienna, Vienna, Austria; 2 Comprehensive Center for Clinical Neurosciences and Mental Health, Medical University of Vienna, Vienna, Austria; 3 Center for Public Health, Department of Epidemiology, Medical University of Vienna, Vienna, Austria; 4 Institute of Psychiatry, Psychology and Neuroscience, King’s College London, London, UK; 5 Department of Psychiatry and Psychotherapy, Faculty of Medicine and University Hospital of Cologne, Cologne, Germany

**Keywords:** alcohol, alcohol use disorder, health, older adults, substance use

## Abstract

**Background:**

The prevalence of alcohol use disorder among older adults is increasing, with this population being particularly vulnerable to alcohol’s detrimental effects. While knowledge of preventative factors is scarce, physical activity has emerged as a potential modifiable protective factor. This study aimed to examine associations between alcohol consumption and physical activity in a large-scale, multi-national prospective study of the older adult population.

**Methods:**

Longitudinal data from the SHARE study on physical activity, alcohol consumption, demographic, socioeconomic, and health variables, were analyzed in older adults. Individual-level data were examined using logistic regression models. Both cross-sectional and longitudinal models were calculated to account for potential latency in the association between physical activity and alcohol consumption.

**Results:**

The study included 3133 participants from 13 countries. Higher physical activity levels were significantly associated with higher alcohol consumption in cross-sectional (*p* = 0.0004) and longitudinal analyses (*p* = 0.0045) over a median follow-up of 6 years. While the presence of depressive symptoms and higher educational attainment were associated with higher alcohol consumption, female sex and persons with lower perceived health showed lower frequency of alcohol consumption. Additionally, the country of residence also proved to be a relevant factor for alcohol consumption.

**Conclusions:**

Higher levels of physical activity showed an association with higher alcohol consumption in older adults. Future research should investigate whether this association is causal and underpinned by neurobiological, social, or methodological factors.

## Background

Over the last decades, the proportion of older (≥ 50 years old) and senior (≥ 65 years old) adults among the total population in the European Union has been increasing. With this trend expected to continue, the proportion of people aged 65 or older is estimated to reach 30% by 2070 [1]. This demographic shift is expected to pose considerable challenges for healthcare and social systems due to the increased burden of multimorbidity and, consequentially, the increased need for long-term care. Of particular concern, mental health issues and the prevalence of psychiatric diagnoses in this population are expected to rise [2].

Notably, hazardous alcohol consumption is becoming increasingly prevalent among older adults: Epidemiological studies have shown rising rates of harmful alcohol consumption and alcohol use disorder (AUD) [3–6]. Several reasons have been hypothesized, such as late-onset AUD occurring due to life-changing events in later life, including retirement, loss of family members, or health issues [7]. Congruously, one-third of older adults with AUD have developed the condition later in life, while two-thirds have already been diagnosed with AUD earlier in life [8]. Furthermore, it is suggested that the social and cultural security afforded today’s older adults (e.g., increases in wealth, increases in quality and availability of healthcare, times of relative security, and little scarcity) have led to a generation that has already had the opportunity of exhibiting regular, and possibly hazardous, alcohol consumption as young adults and now continues this habit in older age [9]. This trend of hazardous alcohol consumption in older age is of high concern as older adults, due to changes in the distribution volume of water and the metabolism of alcohol, exhibit an exceptionally high susceptibility to alcohol-related harm [8, 10]. Thus, even relatively small amounts of alcohol may cause significant detrimental effects on the central nervous system [10]. In addition, alcohol can also interact with the absorption, distribution, and metabolism of drugs, affecting their effectiveness and increasing side effects [11]. Concurrently, the often-present chronic conditions in older adults may be negatively affected by alcohol consumption [12]. The distinct vulnerability of older adults to alcohol-related harm, combined with the increasing prevalence rates of AUD within this population, emphasizes the importance of prevention, therapeutic, and diagnostic measures focused on AUD in older adults.

Previous research suggests that certain risk factors, including socio-economic, socio-demographic, and biographical characteristics, influence individual alcohol consumption patterns [13, 14]. Concerning the group of older adults, for example, specific personality characteristics were also found as risk factors for hazardous alcohol consumption [13]. Regarding targets for prevention, modifiable risk factors are of particular relevancy. One of these modifiable factors previously hypothesized is the level of physical activity. However, available data is limited and inconclusive [15, 16]: The relationship between alcohol use and physical activity is described both as a monotone dose–response relationship (i.e., the level of physical activity increases with alcohol use) and as non-monotonous (i.e., an increase in physical activity was observed with moderate drinking levels, while it decreased with binge or heavy alcohol consumption) [16, 17].

Growing evidence indicates physical activity influences alcohol consumption behavior and may represent a useful adjunct treatment [18, 19]. Therefore, this study aimed to comprehensively examine associations between alcohol consumption and physical activity, considering relevant covariates, by analyzing data from a large-scale, multi-national study of older adults.

## Methods

### Data

For this study, individual-level data from the Survey of Health, Aging and Retirement in Europe (SHARE) was analyzed. SHARE is a longitudinal, cross-national survey that was set up to investigate various questions in many different disciplines, including social sciences, economics, behavior, and health [18]. The survey has been conducted approximately every 2 years since the start of the project in 2004 [19]. Data were collected utilizing computer-assisted personal interviews. As the present study was a retrospective analysis of SHARE data, no ethical approval was required.

Due to limited availability and non-continuous collection of primary variables (alcohol consumption patterns and physical activity levels), only waves two, four, and five were analyzed. The study included data collected in 2006 (Wave 2, DOI: 10.6103/SHARE.w2.800), 2010 (Wave 4, DOI: 10.6103/SHARE.w4.800), and 2013 (Wave 5, DOI: 10.6103/SHARE.w5.800). From a potential baseline population of 35,770 people in Wave 2, participants were selected based on specific criteria. Wave 2 served as the baseline, with either Wave 4 or 5 chosen as the follow-up point, depending on the longest available individual follow-up period.

To improve population homogeneity, several exclusion criteria were applied. Individuals were excluded if they were younger than 50 or older than 59 years at baseline, reported four or more chronic diseases, had severe depressive symptoms (EURO-D-score > 7), or were taking medication for anxiety or depression. Additional exclusions were made for those who refused to answer or had no knowledge of their physical activity level, alcohol consumption, or subjective health perception. Participants with missing values in any regression model variables or lacking follow-up alcohol consumption data (Wave 4 or 5) were also excluded. After applying these criteria, 3133 individuals were included in the final analysis.

### Measures

#### Data on alcohol consumption

The dependent variable, alcohol consumption, was determined by self-reported frequency of consumption over the last 6 months. The responses were classified into the following three groups: Alcohol consumption on more than 3 days a week, less than 3 days a week and not at all – similar to previous studies [20, 21].

#### Data on physical activity

Physical activity levels were surveyed in SHARE by asking separately how often individuals engage in vigorous and moderate physical activities. Examples of both intensity levels were given in the questionnaire: Sports, heavy housework, or a job involving physical labor were indicated as vigorous physical activity, gardening, cleaning the car, or going for a walk as moderate activity. For both, participants stated their frequency by choosing from the options (1) more than once a week, (2) once a week, (3) one to three times a month, (4) hardly ever, or never. For our analysis, we created a composite variable from these two questions according to the following classification: Individuals who reported any frequency of vigorous physical activity were considered as engaging in “vigorous activity,” those with any frequency of moderate physical activity but no vigorous activity as engaging in “moderate activity” and individuals that reported to engage hardly or never in moderate physical activity, were considered as “inactive” – similar to previous studies [22].

#### Data on covariables

Additional covariates used were depressive symptoms, sex, country of living, age at baseline, subjective perception of one’s health status, number of chronic diseases, marital status, and level of education. Depressive symptoms were evaluated with the EURO-Depression-Score (EURO-D), a 12-item scale that was developed for use in a transnational and geriatric context [23]. Furthermore, age (at baseline) was used as a continuous covariate. Subjective health perception was measured by a five-point Likert-Scale ranging from (1) poor to (5) excellent. Regarding the number of chronic diseases, a score was calculated from the sum of (predefined) medically diagnosed diseases. Marital status was classified as done elsewhere [24] into married and not married, with the latter category also including individuals who were divorced, separated, widowed, or never married. The educational status was grouped into low, middle, and high educational levels using the categories of the International Standard Classification of Education Degrees (ISCED 1997) obtained by the SHARE survey as done elsewhere [24].

### Statistics

To examine potential latency in the association between alcohol consumption (dependent variable) and physical activity (independent variable), both cross-sectional and prospective logistic ordinal regression models were calculated. The cross-sectional model analyzed Wave 2 data, incorporating multiple independent variables: physical activity, depression symptoms, sex, age, country, health status, number of chronic diseases, marital status, and education. For the prospective model, the dependent variable was changed from alcohol consumption at Wave 2 to alcohol consumption at either Wave 4 or Wave 5 (using the latest available data). The prospective model included all previously mentioned independent variables plus an additional variable indicating the wave number (4 or 5) of the final data collection point. All analyses were conducted using SAS version 9.4 (SAS Institute Inc., Cary, NC).

## Results

### Study population

The study population consisted of a total of 3133 (54.4% females) participants living in 13 different countries: Austria, Belgium, Czech Republic, Denmark, France, Germany, Israel, Italy, Netherlands, Poland, Spain, Sweden, and Switzerland (see Table 1 for more detailed information of the study population).

**Table 1.** Descriptives of study population

Characteristic	*N*	Percentage (%)	Mean ± Std.dev.	Median
Age at baseline			54.1 **±** 2.9	54
Sex				
Male	1429	45.6		
Female	1704	54.4		
Physical activity level				
Inactive	114	3.6		
Moderate activity	782	24.9		
Vigorous activity	2237	71.4		
Alcohol consumption frequency				
More than 3 days a week	924	29.5		
Less than 3 days a week	1495	47.7		
Not at all	714	22.8		
Euro-Depression-Score			1.79 ± 1.66	1
Number of chronic diseases				
0	1216	38.81		
1	1078	34.41		
2	557	17.78		
3	282	9		
Subjective health perception				
Excellent	457	14.5		
Very good	817	26.1		
Good	1220	38.9		
Fair	523	16.7		
Poor	116	3.7		
Marital status				
Not married	614	19.6		
Married	2519	80.4		
Educational attainment status				
Low	966	30.8		
Middle	1406	44.9		
High	761	24.3		
Country				
Austria	22	0.7		
Germany	222	7.1		
Sweden	170	5.4		
Netherlands	278	8.9		
Spain	201	6.4		
Italy	293	9.4		
France	243	7.8		
Denmark	398	12.7		
Switzerland	210	6.7		
Belgium	126	4.0		
Israel	59	1.9		
Czech Republic	413	13.2		
Poland	498	15.9		

### Cross-sectional model

**Figure 1.**
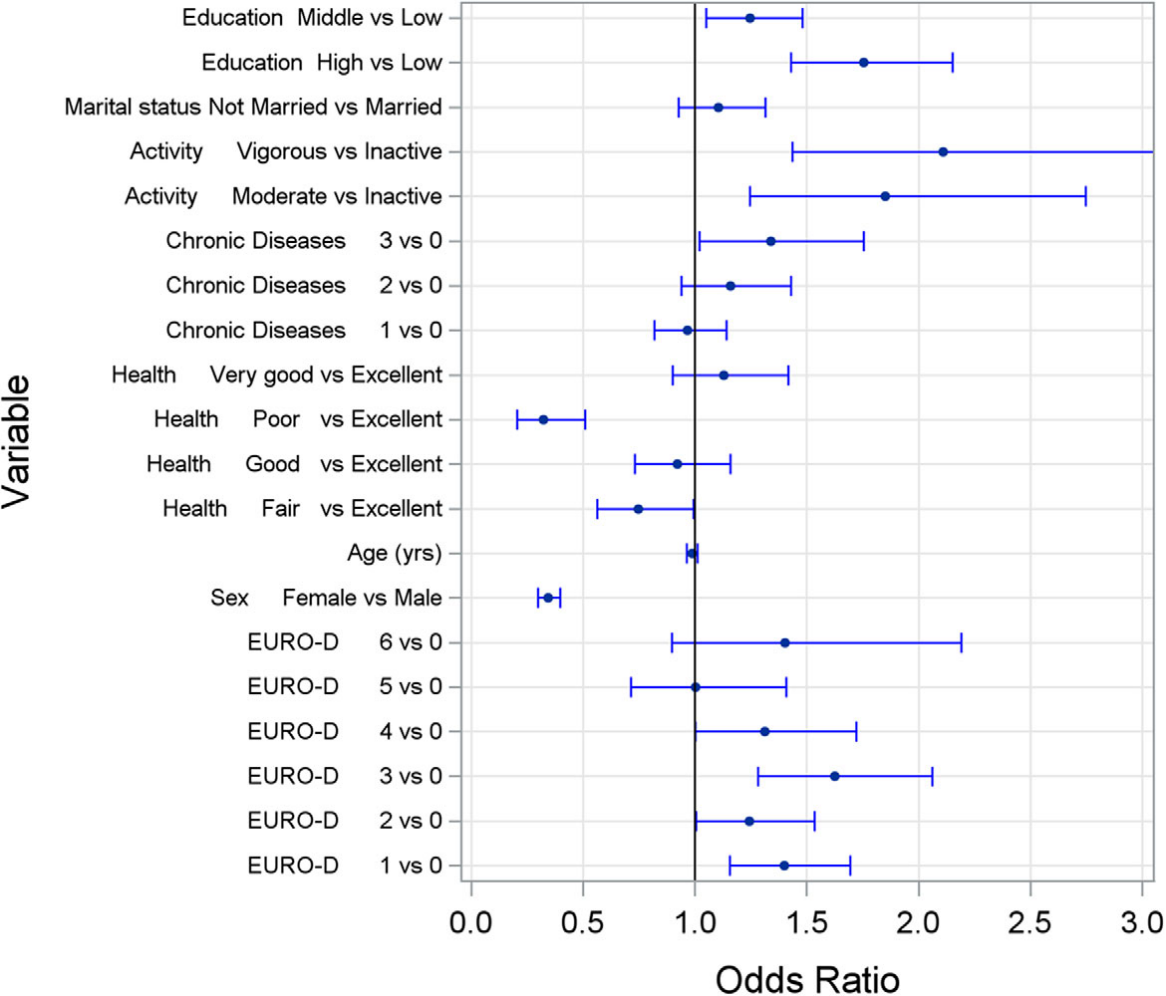
Odds ratios and corresponding 95% confidence intervals for the frequency of alcohol consumption at baseline obtained by a cross-sectional ordinal logistic regression model. Explanation: activity, physical activity. Chronic diseases, number of diagnosed chronic diseases. Health, perceived health. EURO-D, Depression Scale.

In the baseline model examining factors affecting alcohol consumption, physical activity showed a significant positive association with alcohol consumption frequency (*p* = 0.0004; moderate activity: OR = 1.9, 95% CI [1.25–2.75]; vigorous activity: OR = 2.1, 95% CI [1.44–3.10]). Several other factors were significantly associated with more frequent alcohol consumption: male sex (*p* < 0.0001), better subjective health perception (*p* < 0.0001), higher severity of depressive symptoms (*p* = 0.0008), and higher educational attainment (*p* < 0.0001). Country of residence also emerged as a significant factor (*p* < 0.0001). Age, marital status, and number of chronic diseases showed no significant associations. Detailed results are presented in Figure 1 and Supplementary Table S1 in the Supplements.

### Prospective model

**Figure 2.**
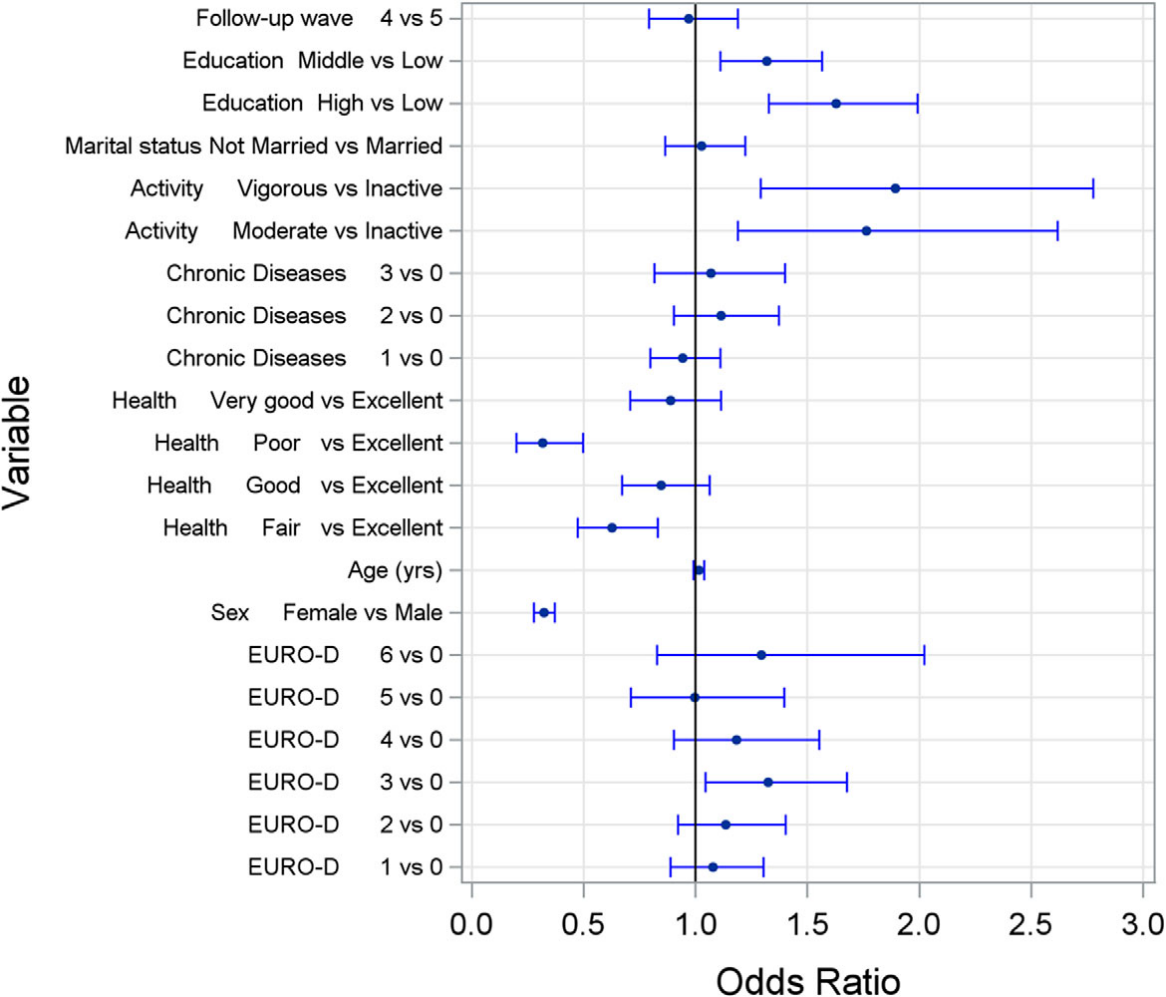
Odds ratios and corresponding 95% confidence intervals for the frequency of alcohol consumption at follow-up obtained by a longitudinal ordinal logistic regression model. Explanation: activity, physical activity. Chronic diseases, number of diagnosed chronic diseases. Health, perceived health. EURO-D, Depression Scale.

The prospective model examined whether baseline risk factors predicted alcohol consumption frequency at follow-up (Wave 4 or 5, median follow-up time 6 years). Consistent with the cross-sectional findings, several baseline factors significantly predicted follow-up alcohol consumption frequency: physical activity level (*p* = 0.0045; moderate activity: OR = 1.8, 95% CI [1.19–2.62]; vigorous activity: OR = 1.9, 95% CI [1.29–2.78]), educational attainment (*p* < 0.0001), perceived subjective health (*p* < 0.0001), and male sex (*p* < 0.0001). Country of residence remained a significant factor (*p* < 0.0001). Unlike the cross-sectional model, depressive symptoms did not significantly predict alcohol consumption at follow-up. These results are visualized in Figure 2.

## Discussion

Our analysis of 3133 older European adults revealed a significant positive association between physical activity levels and alcohol consumption frequency, demonstrated in both cross-sectional and longitudinal models. This relationship persisted after controlling for multiple variables, with educational attainment, country of residence, male sex, and perceived health status also showing significant associations with alcohol consumption patterns. This study represents the first transnational investigation of these relationships, providing novel insights into the complex interplay between physical activity and alcohol consumption behaviors among older adults.

The primary result, the positive correlation between alcohol consumption frequency and physical activity, is consistent with previous studies in older adults [25, 26] and similar results have also been reported in other populations (e.g., college students, representative samples of U.S. population) [27–31]. However, data is still inconclusive and a negative association between alcohol consumption and physical activity has also been postulated in a population of older adults [32] and in the general population [33].

While one might assume that physically active (i.e., health-conscious) individuals would engage in health-promoting behavior and avoid behavior that is harmful to health, the current findings do not support this assumption. Possible explanations for this somewhat counter-intuitive finding in this population include the following:

First, the so-called “*abstainer bias”* may be responsible for masking an actual negative association between physical activity and alcohol consumption, in that a subgroup (particularly older adults of ill health, sometimes in care settings) consumes little or no alcohol due to their poor health [34–36]. These (involuntary) abstinence-maintaining individuals might, at the same time, be too sick to be physically active, which, subsequently, may obscure the association between the two factors for the entire population. This hypothesis is further corroborated by our data, as subjective health perception and alcohol consumption were positively associated (those with a subjectively perceived poor health consumed less alcohol). In contrast to this hypothesis and previous literature [37], no association of the number of chronic diseases diagnosed with alcohol consumption frequency was found in the present sample of older adults. Possible interpretations may be that, by excluding participants with four or more diagnosed chronic diseases for a more homogenous sample, we excluded those who were physically too ill to continue drinking and be physically active. Furthermore, common diagnoses in older adults, such as hypertension, dyslipidemia, and non-insulin-dependent diabetes mellitus in older adults often have little to no impact on their overall well-being and physical fitness [38, 39].

Another possible explanation for the study’s finding of increased frequency of alcohol consumption in those with increased physical activity may be that the “fitter” and more physically active people are, the more they might engage in social activities, possibly also involving alcohol consumption (especially keeping the relatively young age at baseline in mind) [27]. In this case, increased drinking behavior would represent an indirect consequence of increased social participation and a more active lifestyle. In agreement with this theory, Gonzalez-Rubio [26] suggests that the association between physical activity and alcohol might be modulated by specific drinking patterns and the type of predominantly consumed alcoholic beverage. Further studies reported similar results, indicating that individuals with predominant wine consumption were more physically active compared to individuals with predominant consumption of beer or spirits [40, 41].

Another explanation for the found positive association between alcohol consumption and activity levels, postulated by previous literature, is a compensatory increase in physical activity as a way of attempting to reduce the expected negative consequences of alcohol consumption [28, 31]. This hypothesis was developed upon investigations in college students and young adults: Graupensberger et al. [31] found alcohol use and physical activity to not be significantly associated at the between-person level but, at the within-person level, with a lagged effect. In their analysis, alcohol consumption was positively correlated with future (i.e., three months later) physical activity. Importantly, physical activity did not correlate with future alcohol use, which may indicate that the relationship between these two factors is unidirectional. In this context, *French* et al. [27] suggest that individuals may tend to mistakenly believe that proactively engaging in health-promoting behaviors (e.g., physical activity) may alleviate the deleterious effects of ‘unhealthy’ behaviors (e.g., alcohol consumption).

A further hypothesis suggests that persons engaging in a sensation-seeking lifestyle might be more prone to (risky) physical activity and alcohol consumption [27].

Conversely, physical activity has been described as an important moderator of several psychiatric and somatic disorders [42]. A positive effect was found in studies investigating affective disorders [43, 44], but recently, the relevance of physical activity was also investigated for alcohol use disorders [45]. Research in this area ranges from intervention studies for actively drinking patients [46, 47] to epidemiologic studies investigating physical activity as a protective or risk factor for hazardous alcohol consumption.

Besides physical activity associations between alcohol consumption and educational attainment and severity of depressive symptoms were revealed in our study.

The first, although perhaps surprising, is in line with previous research on the population of older adults [13, 48]. A possible explanation is the relationship between educational level and socioeconomic status, which makes alcohol consumption more affordable and may predispose to social drinking [14]. On the other hand, literature also suggests that, despite the higher level of alcohol consumption, a higher level of educational attainment (together with other factors indicating a higher socio-economic status) is associated with a lower risk of alcohol-related consequences and even alcohol-related mortality [49]. As a result, this group could be disproportionately represented in the present study population of older adults.

The second is surprising as individuals with severe depressive symptoms and those taking medication for anxiety or depression had deliberately been excluded from the present analyses. However, it appears that even depressive symptoms below the diagnostic threshold are already associated with increased alcohol consumption.

The significant association between the country of residence and the alcohol consumption frequency of older adults revealed in our analyses is consistent with the previous literature. For instance, research has reported considerable variation in alcohol consumption between countries in the general population as well as the population of older adults specifically [50–52]. These differences may be attributed to socio-cultural differences (e.g., norms, beliefs) and variations in economic factors and alcohol policies (e.g., pricing), as the prevalence of increased alcohol consumption was highest for locations with high socio-demographic index and lowest in locations with low-to-middle socio-demographic index [52]. It is also well known, as replicated in this study, that male sex is associated with increased alcohol consumption, with this correlation being described both cross-nationally and for individual countries [50, 53].

As mentioned above, we found a significant association between sex and alcohol consumption frequency, with women being less likely to drink alcohol compared to men, a finding which is well described in previous literature [54]. The association of sex and alcohol consumption may, besides the biological differences causing a lower tolerance in females, be due to the persistence of cultural norms and social expectations leading to a lower consumption in females as it is seen as less “socially acceptable” [50, 53, 55]. Furthermore, literature postulates that females tend to more often engage in conservative lifestyles and find themselves in more stable social networks than males, which may lead to a health-focused lifestyle [56].

The rising trend of alcohol consumption among older adults suggests a potential underestimation of alcohol’s detrimental health effects in this population. These findings highlight the urgent need to enhance education about alcohol-related risks and consequences specifically tailored for older adults, with particular emphasis on understanding the cumulative health impacts and risk aggregation that make this age group especially vulnerable.

Several limitations of the current study warrant consideration, though they also point to valuable directions for future research. While our retrospective analysis provided robust longitudinal and transnational data, it precluded establishing causality or directionality in the observed associations. Future studies should address this through prospective and, if possible, randomized controlled trials, specifically examining the causal relationship between physical activity and alcohol consumption in older adults. The analysis was also constrained by the variables and operationalizations available in the SHARE survey, particularly regarding alcohol consumption and physical activity measures. The inconsistency of which precluded more extensive analyses of the temporal changes and the interplay of these parameters. The type of assessment of physical activity in the SHARE survey, as well as the chosen categorization of the same in the current study, represent a limitation. It must be noted that the relatively high proportion of participants engaging in vigorous activity could be a consequence of the rather generous definition of vigorous physical activity used or may have been a result of an anticipated desirability when completing the questionnaire. Although a more detailed survey would enable a more precise analysis, it would also pose a challenge for statistical interpretation. To address this, future research should incorporate more comprehensive and objective measures, including daily diary methods, biomarkers for alcohol consumption, accelerometer data for physical activity, and multiple assessment methods to minimize self-reporting bias. Despite these limitations, our study contributes significantly to understanding the relationship between factors associated with alcohol consumption in older adults, providing a foundation for more targeted methodological approaches in future research.

## Conclusions

The primary aim of this study was to determine the association of physical activity with alcohol consumption in older adults by analyzing longitudinal data of a large study population and considering further potential moderators of this association. In correspondence with previous literature, a positive association between physical activity and alcohol consumption could be found in this European population of older adults. The so-called “abstainer-bias,” sensation-seeking lifestyles, “social alcohol consumption” associated with physical activities, and the attempt to compensate for risk through alcohol consumption by increased physical activity are possible explanatory models for this finding. In conclusion, this study confirms that – at least in the case of alcohol consumption – there is not necessarily a direct link between several health-damaging (or health-promoting) behaviors. This finding should be considered when developing screening and interventions for behavioral risk factors in older adults.

## Supporting information

Weber et al. supplementary material Weber et al. supplementary material

## Data Availability

The data that support the findings of this study are available from SHARE-ERIC. Restrictions apply to the availability of these data, which were used under license for this study. Data are available at http://www.share-project.org with the permission of SHARE-ERIC.

## Supplementary material

The supplementary material for this article can be found at http://doi.org/10.1192/j.eurpsy.2025.2417.
