# Supplementary material for: Physical activity in older adults as a predictor of alcohol consumption – a longitudinal analysis of 3133 individuals in the SHARE study
Source: Eur Psychiatry. 2025 Mar 20;68(1):e47. doi: 10.1192/j.eurpsy.2025.2417 (PMC12041726; doi:10.1192/j.eurpsy.2025.2417)
Supplement: Weber et al. supplementary material [file S0924933825024174sup001.pdf]

Cross-sectional logistic regression model

Effect	Point Estimate	95% Wald Confidence Limits		
1 vs 0 EURO-Depression-Score	**1.399**	**1.155**	**1.695**	**p = 0.0008**
2 vs 0 EURO-Depression-Score	**1.244**	**1.007**	**1.536**	
3 vs 0 EURO-Depression-Score	**1.625**	**1.281**	**2.060**	
4 vs 0 EURO-Depression-Score	**1.313**	**1.002**	**1.722**	
5 vs 0 EURO-Depression-Score	**1.003**	**0.715**	**1.408**	
6 vs 0 EURO-Depression-Score	**1.402**	**0.896**	**2.192**	
Female vs Male sex	**0.345**	**0.298**	**0.399**	**p < 0.0001**
Austria vs Poland	**2.362**	**1.035**	**5.391**	**p < 0.0001**
Belgium vs Poland	**2.857**	**1.926**	**4.237**	
Czech Republic vs Poland	**1.664**	**1.274**	**2.173**	
Denmark vs Poland	**3.786**	**2.842**	**5.043**	
France vs Poland	**2.755**	**2.029**	**3.740**	
Germany vs Poland	**2.258**	**1.651**	**3.088**	
Israel vs Poland	**0.246**	**0.136**	**0.443**	
Italy vs Poland	**1.864**	**1.392**	**2.495**	
Netherlands vs Poland	**4.693**	**3.466**	**6.356**	
Spain vs Poland	**1.568**	**1.126**	**2.184**	
Sweden vs Poland	**1.604**	**1.133**	**2.270**	
Switzerland vs Poland	**3.034**	**2.188**	**4.207**	
Age at baseline	0.988	0.965	1.012	p = 0.3385
Fair vs Excellent subjective health perception	**0.748**	**0.564**	**0.993**	**p < 0.0001**
Good vs Excellent subjective health perception	**0.920**	**0.731**	**1.158**	
Poor vs Excellent subjective health perception	**0.323**	**0.205**	**0.509**	
Very good vs Excellent subjective health perception	**1.129**	**0.899**	**1.418**	
1 vs 0 Chronic diseases	0.966	0.818	1.140	p = 0.0560
2 vs 0 Chronic diseases	1.159	0.940	1.429	
3 vs 0 Chronic diseases	1.339	1.021	1.756	
Moderate activity vs Inactive	**1.850**	**1.246**	**2.747**	**p = 0.0004**
Vigorous activity vs Inactive	**2.109**	**1.436**	**3.097**	
Marital status	0.905	0.760	1.077	p = 0.2609
High vs Low educational attainment	**1.754**	**1.431**	**2.151**	**p < 0.0001**
Middle vs Low educational attainment	**1.248**	**1.052**	**1.480**	

Table S1: Odds ratio estimates of the cross-sectional logistic ordinal regression model

Prospective logistic regression model

Odds Ratio Estimates				
**Effect**	**Point Estimate**	**95% Wald Confidence Limits**		
1 vs 0 EURO-Depression-Score	1.077	0.89	1.303	p = 0.3264
2 vs 0 EURO-Depression-Score	1.136	0.92	1.403	
3 vs 0 EURO-Depression-Score	1.325	1.046	1.678	
4 vs 0 EURO-Depression-Score	1.184	0.904	1.552	
5 vs 0 EURO-Depression-Score	0.996	0.711	1.396	
6 vs 0 EURO-Depression-Score	1.294	0.828	2.022	
Female vs Male sex	**0.322**	**0.279**	**0.373**	**p < 0.0001**
Austria vs Poland	**3.136**	**1.353**	**7.270**	**p < 0.0001**
Belgium vs Poland	**4.833**	**3.136**	**7.447**	
Czech Republic vs Poland	**2.068**	**1.507**	**2.836**	
Denmark vs Poland	**3.952**	**2.816**	**5.546**	
France vs Poland	**3.878**	**2.748**	**5.472**	
Germany vs Poland	**2.348**	**1.655**	**3.332**	
Israel vs Poland	**0.314**	**0.168**	**0.585**	
Italy vs Poland	**1.799**	**1.286**	**2.516**	
Netherlands vs Poland	**5.580**	**3.937**	**7.909**	
Spain vs Poland	**2.158**	**1.487**	**3.132**	
Sweden vs Poland	**2.030**	**1.386**	**2.973**	
Switzerland vs Poland	**3.926**	**2.698**	**5.713**	
Age at baseline	1.014	0.990	1.039	p = 0,2393
Fair vs Excellent subjective health perception	**0.627**	**0.473**	**0.832**	**p < 0.0001**
Good vs Excellent subjective health perception	**0.845**	**0.672**	**1.064**	
Poor vs Excellent subjective health perception	**0.316**	**0.200**	**0.498**	
Very good vs Excellent subjective health perception	**0.888**	**0.707**	**1.114**	
1 vs 0 Chronic diseases	0.943	0.799	1.112	p = 0.4080
2 vs 0 Chronic diseases	1.114	0.904	1.373	
3 vs 0 Chronic diseases	1.069	0.816	1.401	
Moderate activity vs Inactive	**1.765**	**1.190**	**2.617**	**p = 0.0045**
Vigorous activity vs Inactive	**1.893**	**1.291**	**2.777**	
Marital status	1.028	0.864	1.223	p = 0.7574
High vs Low educational attainment	**1.627**	**1.328**	**1.993**	**p < 0.0001**
Middle vs Low educational attainment	**1.319**	**1.112**	**1.564**	
Follow-up at wave 4 vs wave 5	0.971	0.792	1.191	p = 0.7779

Table S2: Odds ratio estimates of the prospective logistic ordinal regression model
